# A novel assay for high-throughput screening of anti-Alzheimer’s disease drugs to determine their efficacy by real-time monitoring of changes in PC12 cell proliferation

**DOI:** 10.3892/ijmm.2013.1608

**Published:** 2013-12-27

**Authors:** XUE-QIN HOU, RONG YAN, CONG YANG, LEI ZHANG, RU-YU SU, SI-JUN LIU, SHI-JIE ZHANG, WEN-QING HE, SHU-HUAN FANG, SHU-YI CHENG, ZI-REN SU, YUN-BO CHEN, QI WANG

**Affiliations:** Institute of Clinical Pharmacology, Guangzhou University of Chinese Medicine, Guangzhou 510405, P.R. China

**Keywords:** anti-Alzheimer’s disease drugs, PC12 cells, Chinese herbal compound, pharmacological, cell index, real-time cell monitoring device

## Abstract

Alzheimer’s disease (AD) is a neurodegenerative disease that is characterized by the accumulation of senile plaque and neurofibrilary tangle formation in the brain, including the cerebral cortex and hippocampus. Nowadays, the first-line treatment for AD is the application of acetylcholinesterase inhibitors. However, acetylcholinesterase inhibitors are basically anti-symptomatic for a limited aspect of AD pathology and are associated with serious side-effects. With the advantage of multiple targets, pathways and systems, Chinese herbal compounds hold promising potential for the development of drugs for the treatment of AD. Over the past few years, with the development of Chinese herbal compounds and *in vitro* pharmacological studies, cell-based disease models are one of the main methods used to screen Chinese herbal compounds for potential efficacy. Testing the efficacy of possible anti-Alzheimer’s disease drugs and the development of new drugs are hindered by the lack of objective high-throughput screening methods. Currently, the assessment of the effects of drugs is usually made by MTT assays, involving laborious, subjective, low-throughput methods. Herein, we suggest a novel application for a real-time cell monitoring device (xCELLigence) that can simply and objectively assess the effective composition of Chinese herbal compounds by assessing amyloid-β peptide Aβ1-42-induced apoptosis in PC12 cells. We detected the proliferation and motility of the cells using a fully automated high-throughput and real-time system. We quantitatively assessed cell motility and determined the real-time IC_50_ values of various anti-AD drugs that intervene in several developmental stages of Aβ1-42-induced apoptosis in PC12 cells, Then, we identified the optimal time phase by curative efficacy. Our data indicate that this technique may aid in the discovery and development of novel anti-Alzheimer’s disease drugs. It is possible to utilize a similar technique to measure changes in electrical impedance as cells attach and spread in a culture dish covered with a gold microelectrode array that covers approximately 80% of the area on the bottom of a well. As cells attach and spread on the electrode surface, it leads to an increase in electrical impedance of 9–12. The impedance is displayed as a dimensionless parameter termed the cell index, which is directly proportional to the total area of tissue culture well that is covered by the cells. Hence, the cell index can be used to monitor cell adhesion, spreading, morphological variation and cell density.

## Introduction

Along with the increasing average life-span of humans over the past decades, human society is becoming more concerned about the malfunctions associated with aging (1). Senile dementia is one of the most formidable consequences of aging, including Alzheimer’s disease (AD), which accounts for >50% of senile dementia (2). AD is characterized by neuronal loss and the presence of extracellular senile plaques mainly constituted by amyloid-β peptide (Aβ). Bu-Shen-Yi-Zhi prescription (BSYZ) is a traditional Chinese compound prescription which is commonly used in China for the treatment of AD. Due to its complex components, it is difficult to conduct studies on its curative mechanisms. Traditional methods for screening drug components involve a great amount of effort with high development expenses and limited economic return. Furthermore, a usually overlooked impediment to drug development is the lack of objective high-throughput screening methods for assessing drug efficacy. In this study, we investigated the effects of different effective components of BSYZ compound on Aβ1-42-induced apoptosis in PC12 cells. By applying real-time cellular analysis (RTCA) for screening the main active ingredients of BSYZ, we found that RTCA technology with biological and pharmacological relevance amenable for high-throughput screening, is a novel tool that can by applied for high-throughput screening. The Bu-Shen-Yi-Zhi compound contains *Fructus Cnidii* (FC), *Panax ginseng*, *Cortex Moutan*, *Polygonum multiflorum*, *Fructus lycii* and *Ligustrum lucidum (*Ait. Patent no. ZL 200610112916.1).

## Materials and methods

### Real-time cell electronic sensing (RT-CES) system

**Figure 1:**
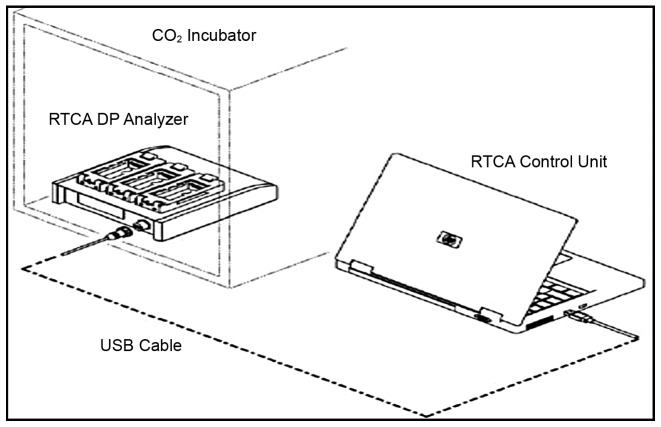
Real-time cell electronic sensing (RT-CES) system.

The RT-CES system (RTCA; Roche Applied Science, Mannheim, Germany) used in this study consists of single-use E-plates inserted into an RTCA single-plate (SP) station which is located within the incubator. It is comprised of three components: an electronic sensor analyzer, a device station and a 16-well strip (Fig. 1). Giaever and Keese first described a technique for measuring fluctuations in impedance when a population of cells grow on the surface of electrodes (3,4). We utilized a similar technique to measure changes in electrical impedance as cells attach and spread in a culture dish covered with a gold microelectrode array that covers approximately 80% of the area on the bottom of a well. Under the RT-CES software control, the sensor analyzer can automatically select wells to be measured and continuously conduct measurements on wells.

### Cell culture

The PC12 cells were obtained from the Shanghai Institutes for Biological Sciences, PC12 cells were grown in DMEM medium containing 10% heat-inactivated horse serum and 5% FBS at 37°C in a humidified atmosphere of 5% CO_2_/95% air.

### Deployment of RT-CES in cell culture

The xCELLigence system used in this study consists of single-use E-plates inserted into an RTCA single-plate station which is located within the incubator. All steps were performed under sterile conditions. The cells were passaged 1 day before the experiments until they reached 60–80% confluence. The cells were then trypsinized by the addition of 0.05% Trypsin/0.02% EDTA solution at room temperature or 37°C for 1–2 min. Trypsinization was terminated by the addition of 10% FBS-containing medium. Subsequently, 100 μl cell suspension of 5×10^4^ cells/well were added to the CIM-Plate 16, and the CIM-Plate 16 was loaded onto the RTCA DP Analyzer inside the incubator for measurements to be taken.

### Preparation of BSYZ extracts

BSYZ prescription compound was chopped and extracted with distilled water at 80°C for 2 h. This procedure was repeated three times. After filtering, the mixture was concentrated under reduced pressure using a rotary evaporator to afford 50 g (1 g/ml) of the crude water extract (WE). The BSYZ compound was extracted with ethanol (85% v/v) for 2 h with occasional mechanical shaking, and the extraction process was repeated, and the mixture was then concentrated under reduced pressure using a rotary evaporator to afford 50 g (2 g/ml) of the crude ethanol extract (EE).

Eight samples (numbered 1–8) were extracted from the former two extractions using petroleum ether, dichloromethane, ethyl acetate and n-butanol in this order. The crude extract was concentrated under a vacuum and evaporated to dryness to yield a brown, sticky fraction (F1, F2, F3, F4, F5, F6, F7 and F8; 5 g/ml) (5). The crude extract and its fractions were stored in a refrigerator until use.

### Preparation of aged Aβ1-42

Aβ1-42 was solubilized in DMEM at 1 mm, incubated in a capped vial at 37°C for 4 days to form aggregates (6), and stored at 20°C until further use. For the aging procedure, the stock solutions were diluted to the desired concentration (200, 100, 50, 25 and 12.5 μM) immediately prior to use and added to the culture medium.

### Toxicity test of Aβ1-42

The PC12 cells were cultivated in 16-well plates at a density of 5×10^4^ cells/well. After adhesion for 24 h, the medium was replaced with serum-free medium at 200 μl per well. Aβ1-42 was added at the indicated concentrations, unless otherwise specified. The cells were first stabilized at 37°C for 24 h with culture medium. Thereafter, the culture medium was replaced with fresh serum-free DMEM with or without various concentrations of Aβ1-42 (final concentrations: 12.5, 25, 50, 100 and 200 μM) for 24 h.

### Toxicity test of BSYZ extracts and fractions

The PC12 cells were seeded on a 16-well plate at a density of 5×10^4^ cells/well. After adhesion for 24 h, the medium was replaced with serum-free medium at 200 μl per well. The culture medium was replaced with fresh serum-free DMEM with or without various concentrations of BSYZ extracts and fractions (final concentrations: 1.5625, 3.125, 6.25, 12.5, 25, 50 and 100 μg/ml) for 24 h. There were eight fractions: 100 mg/ml stock solution was prepared by the addition of 10 mg crude extract into 0.1 ml DMSO and dissolving by an ultrasonic dissolving instrument (ETUS). The working solution was then prepared by diluting the stock solution with serum-free DMEM.

### Effect of BSYZ extracts against the damaging effects of Aβ1-42

The PC12 cells were seeded onto a 16-well plate at a density of 5×10^4^ cells/well. After complete adhesion for 24 h, the medium was replaced with serum-free medium at 200 μl per well. BSYZ extracts were added at the indicated concentrations. The cultivation was continued for an additional 24 h. Subsequently, 50 μM of Aβ1-42 were added to the cells followed by incubation for another 24 h. The control cells were treated in a similar manner without the addition of BSYZ extracts and Aβ1-42 to the serum-free culture medium. The cultivation was continued.

### Statistical analysis

Parametric values were compared by the two-tailed, unequal variance Student’s t-test using a minimum of four replicate wells. The mean absolute percentage error (MAPE) was calculated as the average of the absolute value of the percentage difference between the test and reference well CI values over the duration of the experiment.

## Results

### Cell quantification and monitoring proliferation

**Figure 2:**
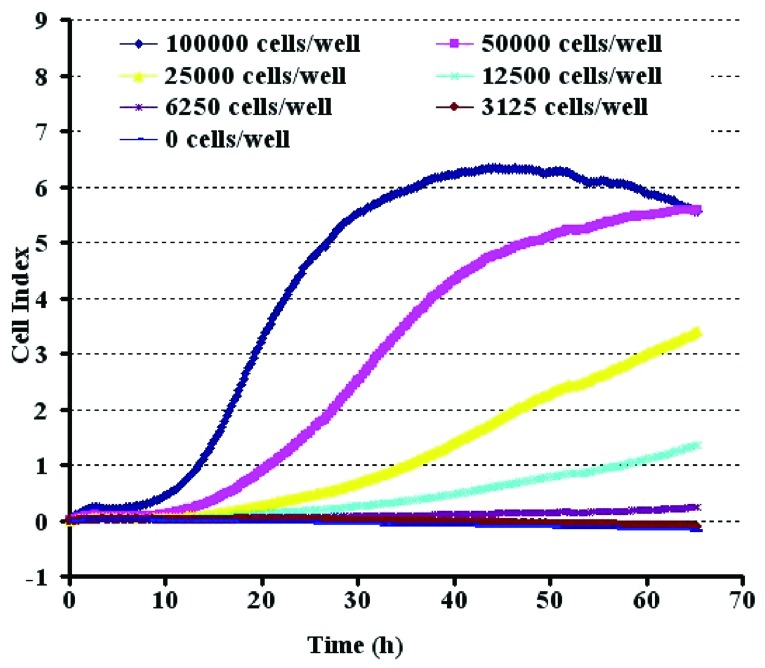
Cell quantification on the real-time cell electronic sensing (RT-CES) system. The top panels show the RT-CES growth curves of PC12 cells at different starting numbers ranging from 3,125 to 100,000. The slope of the growth curve indicates the different cell-specific growth rates. The optimal inoculation density is 50,000 cells per well.

The CI values determined on the RT-CES system were linearly correlated with the PC12 cell numbers over a range of 3,125 to 100,000 cells (Fig. 2). To evaluate the precision in the RT-CES assay, the CI values for each cell number were measured by three replicates of appropriate cell numbers. The cells were seeded at 50,000 cells/well onto an E-Plate, which was the optimal inoculation density.

### Effect of Aβ1-42 on cell viability

**Figure 3:**
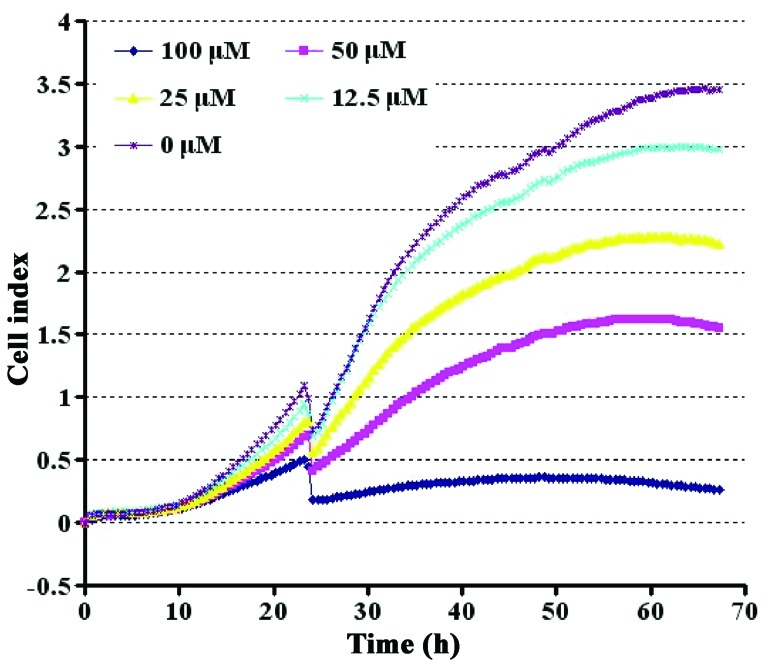
Dynamic monitoring of cytotoxic response to Aβ1-42. PC12 cells were treated with Aβ1-42 at concentrations of 100, 50, 25 and 12.5 μM in serum-free medium. At the concentration of 50 μM, Aβ1-42 reduced the viability of PC12 cells to approximately 55%.

To determine whether the CI value obtained on the RT-CES system quantitatively correlated with the PC12 cell numbers, the PC12 cells were titrated and grown on the sensor devices, resulting in device impedance signals and were accurately measured by the RT-CES system. Aβ1-42 suppressed cell viability in a dose-dependent manner. During the initial 48 h, cell growth was differentially stimulated. However, a prominent suppressive effect appeared at 60 h. At the concentration of 50 μM, Aβ1-42 reduced the viability of the PC12 cells to approximately 50% (Fig. 3).

### Effect of BSYZ extracts on cell viability

**Figure 4:**
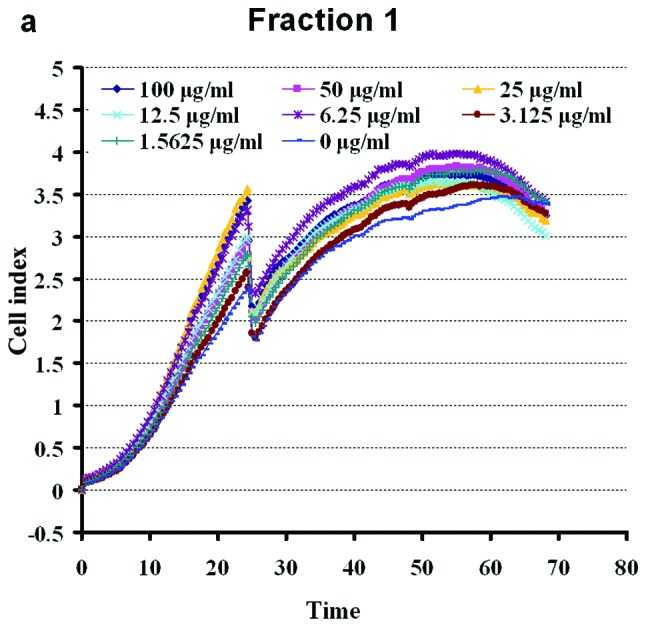

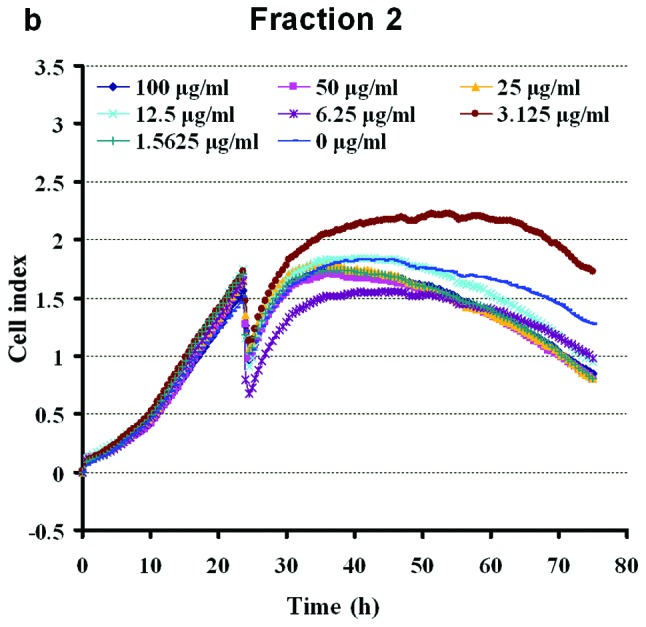

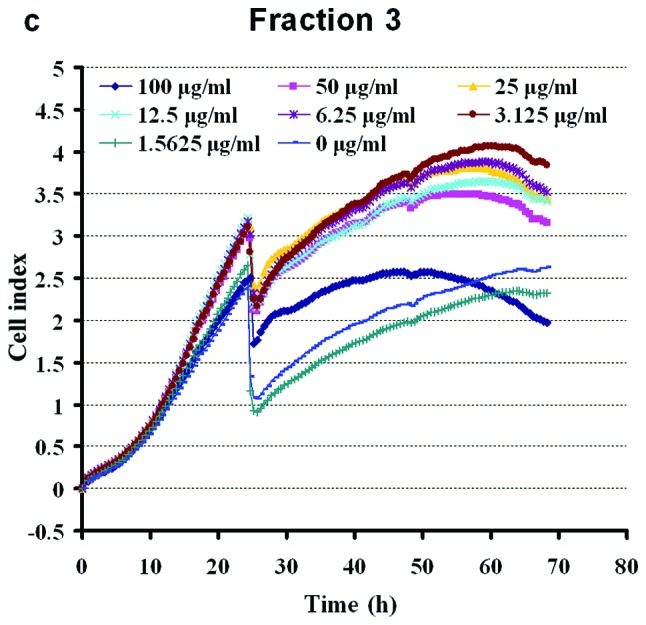

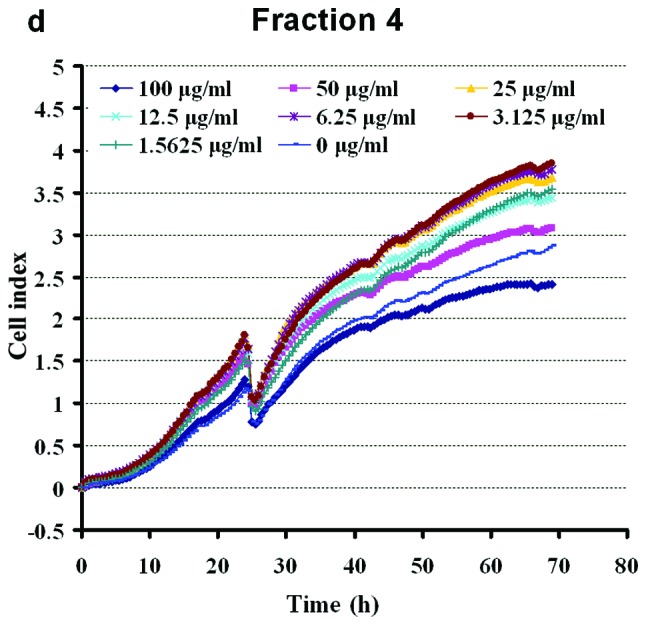

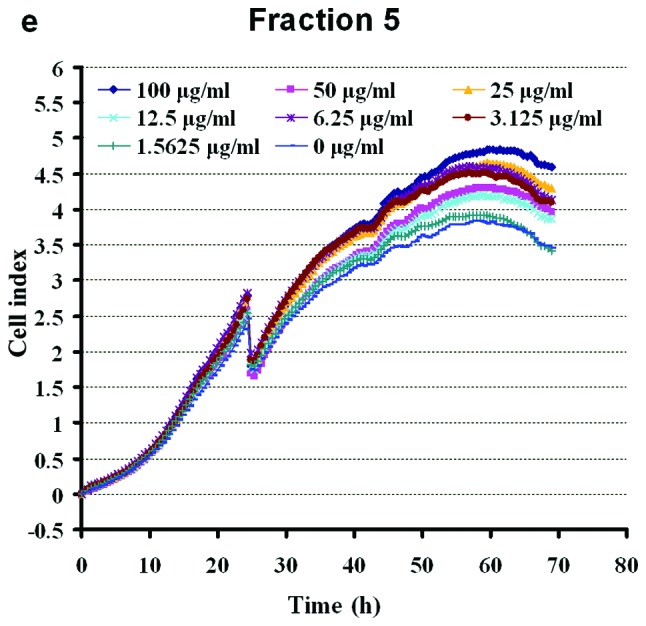

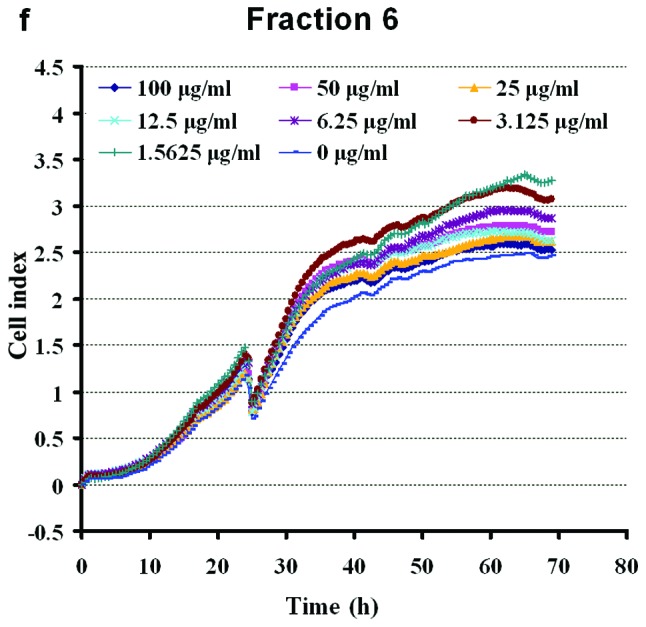

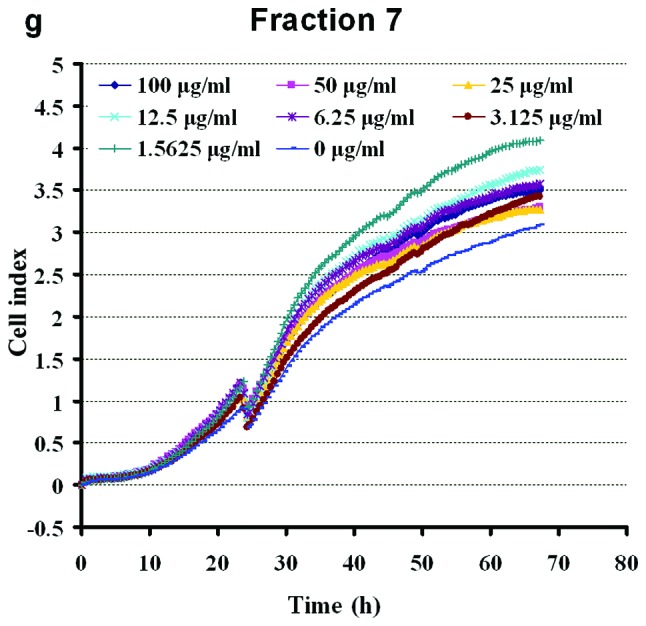

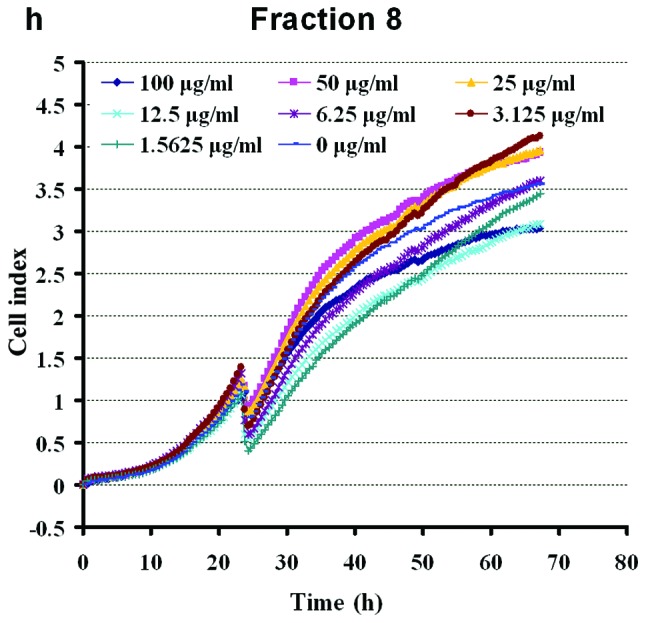
Toxicity of the eight fractions of Bu-Shen-Yi-Zhi prescription (BSYZ) on PC12 cells. (a–h) PC12 cells were treated with eight fractions of BSYZ at concentrations of 100, 50, 25, 12.5, 6.25, 3.125 and 1.5625 μg/ml in serum-free medium for 48 h.

Extracts of eight fractions of BSYZ stimulated the proliferation of PC12 cells in a dose-dependent manner. The cells were shown to grow steadily within the dose range of 100-1.5625 μg/ml (apart from, F2). Doses of 100 μg/ml of F4 and F5 exerted slight inhibitory effect on cell viability (Fig. 4). The results revealed a non-dose-dependent effect on the proliferation of PC12 cells by WE petroleum ether, dichloromethane, ethylacetate and n-butanol fractions in early extraction. At a dose of 100-3.125 μg/ml, F3 enhanced cell viability. The aqueous fraction, F3, was the most effective of the four aqueous fractions, inducing a growth rate of 16.18–60.18%. The effects of the crude ethanol extracts (F6–F7) were significant at 100-1.5625 μg/ml (Fig. 4). The n-butanol fraction, F8, was the least effective of the four ethanol fractions, enhancing the viability of the PC12 cells to approximately 0–40.14%. The ethyl acetate fraction (F7) had intermediate activity, inducing significant (P<0.05) proliferation of PC12 cells at a dose of 1.5625 μg/ml (44.71%). The chloroform and ethyl acetate fractions (F3, F6 and F7) possessed marked PC12 cell stimulating activity, as shown in Fig. 4.

### Effect of BSYZ extracts on Aβ1-42-induced cytotoxicity

**Figure 5:**
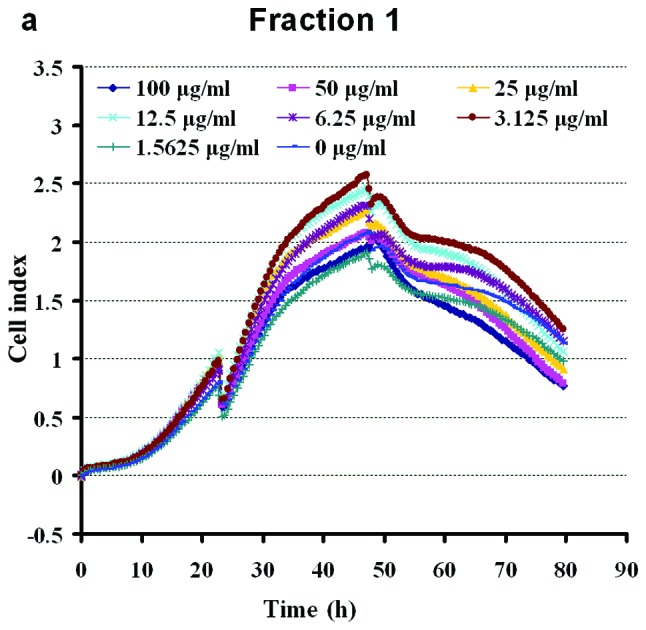

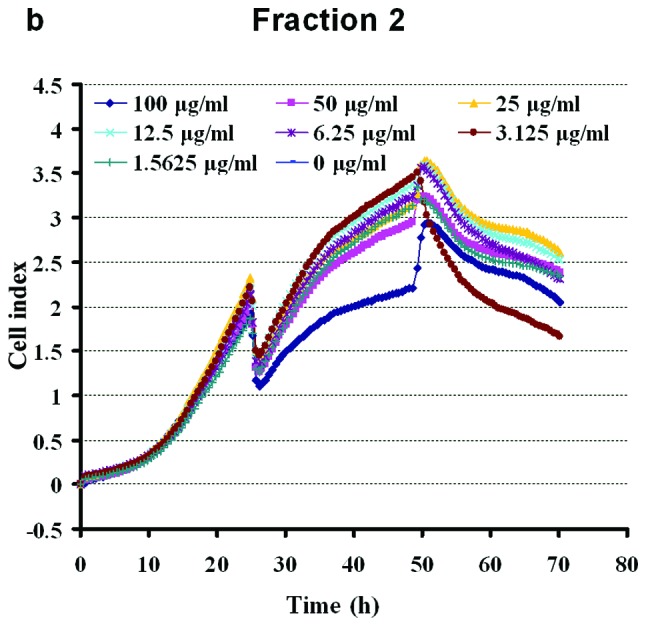

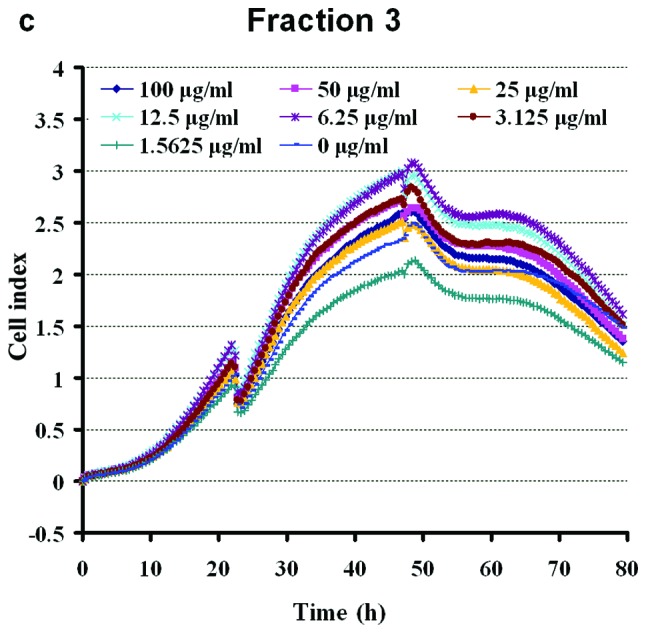

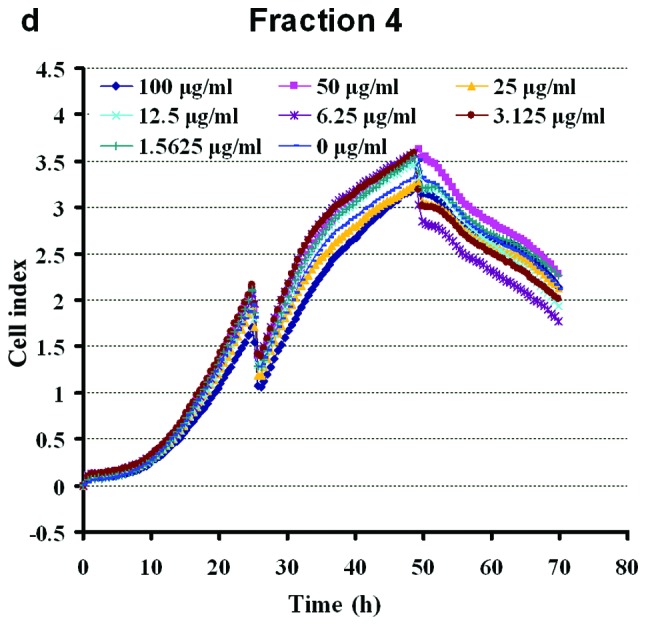

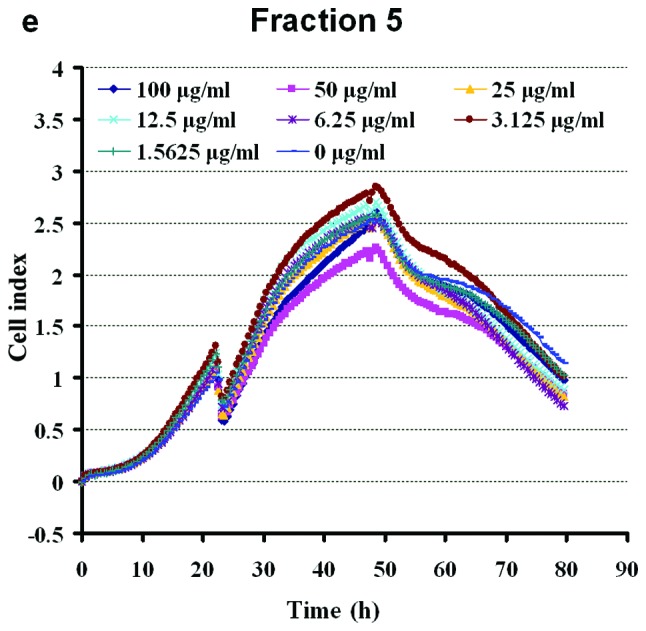

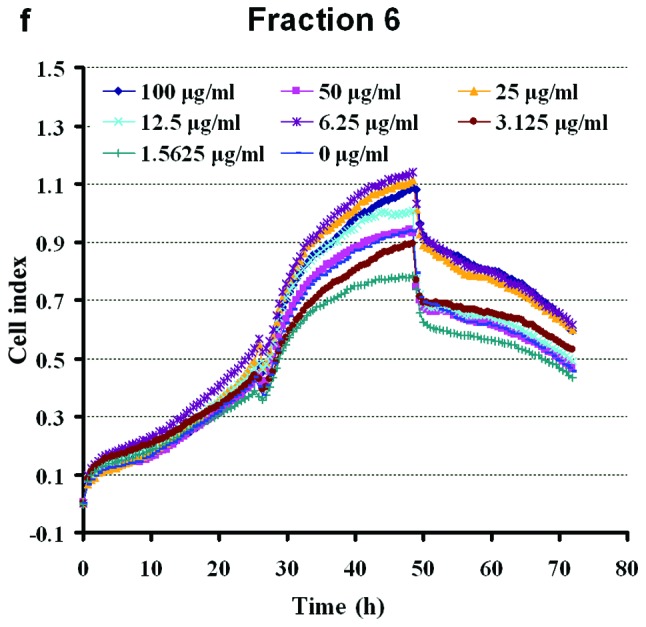

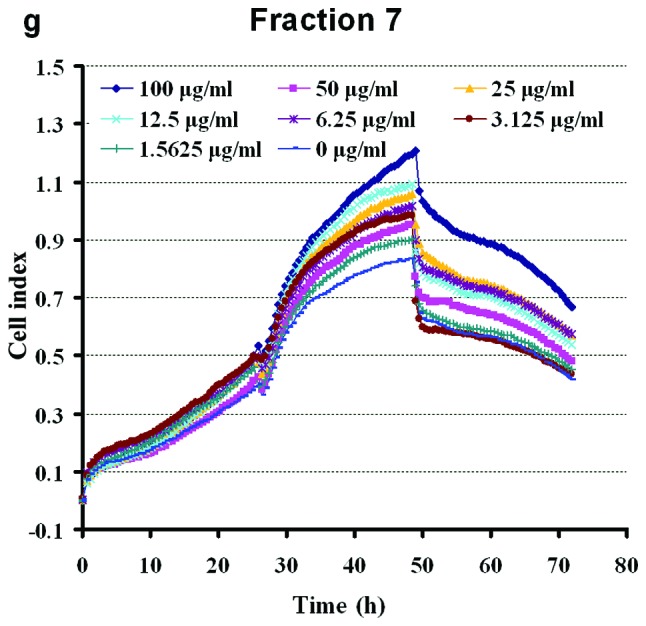

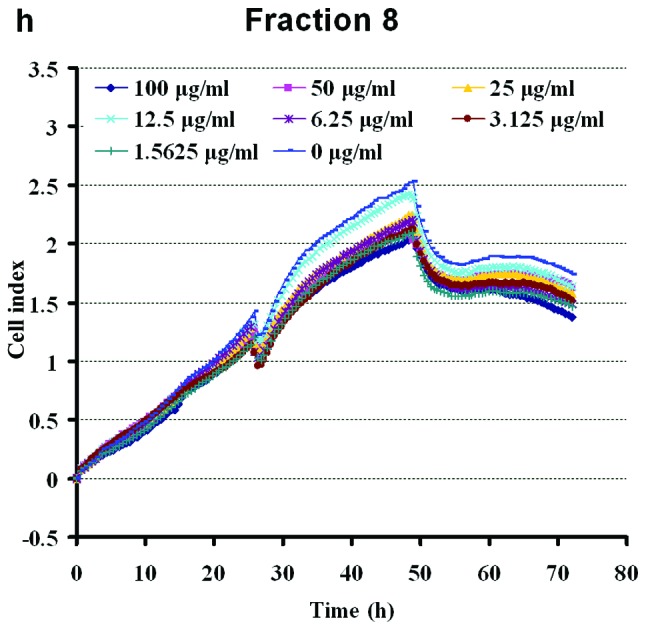
Protective effects of eight fractions of Bu-Shen-Yi-Zhi prescription (BSYZ) against Aβ1-42-induced toxicity in PC12 cells. BSYZ extracts at concentrations of 100, 50, 25, 12.5, 6.25, 3.125 and 1.5625 μg/ml was tested to examine its protective effects against Aβ1-42-induced toxicity at 50 μM. The time intervals for induction were 24 h. Data are the means ± SD of triplicate experiments compared with the group treated with Aβ1-42 for 24 h.

Following incubation for 48 h, the extracts of eight fractions of BSYZ completely reversed the suppressive effect of Aβ1-42 on cell viability (Fig. 5). The results revealed a non-dose-dependent effect on the proliferation of PC12 cells by WE and petroleum ether, dichloromethane ethyl acetate and n-butanol fractions. PC12 cells were pre-treated with BSYZ extracts for 24 h prior to incubation with or without 50 μM Aβ1-42 for another 24 h. At a dose of 100-3.125 μg/ml, F3 enhanced cell viability. The growth rate was 15.18–50.8%. The effect of the crude ethanol extract (F6 and F7) was significant at a dose of 100-3.125 μg/ml (Fig. 5) as compared to the vehicle-treated control group. Under the same conditions, pre-treatment of the cells for 24 h with F6 and F7 at concentrations of 100 and 3.125 μg/ml markedly enhanced cell proliferation (47% and 54%). Although 50 μg/ml of the BSYZ extracts was able to reduce Aβ1-42-induced cell death, no statistically significant differences were observed when compared with the Aβ1-42-treated control group. As shown in Fig. 5, incubation of the PC12 cells with 1.5625 μg/ml for 24 h markedly decreased the cell viability as compared to the control group. The effects of all the fractions on cell proliferation were evaluated to further investigate the protective effects of BSYZ extracts.

## Discussion

PC12 is a cell line derived obtained from a pheochromocytoma of the rat adrenal medulla. PC12 cells have the same characteristics as neuroendocrine cells and have the ability to passage. Therefore, PC12 cells are widely used in neurophysiological and neuropharmacological studies. Previous studies have demonstrated that amyloid fibrils induce PC12 cell death through apoptosis (7,8). Our study first demonstrated the neuroprotective effects of BSYZ extracts on Aβ1-42-damaged PC12 cells, a typical model of AD in a culture system evidenced by increased cell viability and decreased cell apoptosis. In recent decades, attention has been paid to finding natural compounds with advantages of having anti-apoptotic activity and low toxicity for use as neuroprotective agents (9,10). Eight fractions, major active components isolated from BSYZ, have been commonly used as a safe and effective medication ingredient in China for acute kidney-reinforcing for centuries (11–15). Over the past few years, BSYZ has been reported to exert neuroprotective effects *in vivo* and *in vitro* (16–20).

As previously described, the E-plates contain 16 wells in a standard microtiter plate format, with up to 16 wells being monitored at any one time. The ease of experimentation enables the simultaneous monitoring of different fractions or developmental stages on the same plate. The RTCA unit that we used was the original single-plate xCELLigence model (RTCA SP instrument), allowing for the testing of additional samples with PC12 cells per well. These larger scale applications may be adapted to incorporate robotic handling for screening Chinese herbal compounds for their efficacy.

We firstly established the optimum growth of PC12 cells on the microelectrodes in the microwells and evaluated the linear correlation between the cell index and cell numbers without particle treatment in order to develop a quantitative measurement of cell response to particles. PC12 cells have previously been shown to be more sensitive to Aβ1-42. As shown in Fig. 2, the sensing curves of cell index versus incubation time, demonstrating typical cell growth curves of PC12 cells with initial seeding cell numbers approximately 50,000 cells per well under optimized conditions for the RT-CES experiments. At time zero, no cells are attached to the microelectrodes, thus the cell index is zero. With increasing numbers of cells attaching to the microelectrodes over time, the cell index increased. During the log phase of the cell growth, Aβ1-42 suppressed PC12 cell viability in a dose-dependent manner. As shown in Fig. 3, approximately half of the viability suppression was achieved with treatment at a dose of 50 mM Aβ1-42; hence, the 50% lethal dose value of Aβ1-42 was estimated to be 50 μM. This dose was thus selected for use in the following experiments. BSYZ extracts, at a dose within the range of 50-1.5625 μg/ml, had significant effects on cell viability, stimulating PC12 cell proliferation. At doses >50 μg/ml, the cell viability was slightly suppressed (Fig. 4). As shown in Fig. 5, BSYZ exerted a non-dose-dependent on the proliferation of PC12 cells. The effects of the drug were inconsistent with those shown in Fig. 4.

The approach applied in this study has the advantages of easy handling, high sensitivity, real-time monitoring and is thus useful for screening drugs. This approach provides information not only on the onset time a drug or chemical acts on cells, but also information on cell physiology. Thus, we expect to identify the cytotoxicities at different stages for various BSYZ extracts concentrations. In order to determine the status of PC12 cells upon co-incubation with Chinese herbal compounds, the cell cycle and cell apoptosis were examined using the RT-CES system. The time phase of the cell cycle and cell apoptosis analysis reported in our study focused on 64 samples of crude extract of BSYZ (Figs. 4 and 5). Although the RT-CES system can be used for the detection of previously unexpected effects, it is a rather unspecific method and the conclusions about the nature and/or extent of an observed effect can be drawn if the relevant molecular mechanisms associated with the tested samples are investigated simultaneously. In conclusion, we present a novel use of a RTCA device (xCELLigence) that can simply and objectively assess the effectiveness of anti-AD drugs in real-time by measuring motility in a high-throughput, reproducible manner with minimal effort and training required.

## References

[1] Li XL, Wang de S, Zhao BQ, Li Q (2008). Effects of Chinese herbal medicine fuzhisan on aged rats. Exp Gerontol.

[2] Holliday R (1996). The urgency of research on ageing. Bioessays.

[3] Giaever I, Keese CR (1984). Monitoring fibroblast behavior in tissue culture with an applied electric field. Proc Natl Acad Sci USA.

[4] Giaever I, Keese CR (1991). Micromotion of mammalian cells measured electrically. Proc Natl Acad Sci USA.

[5] Ifeoma O, Samuel O, Itohan AM, Adeola SO (2013). Isolation, fractionation and evaluation of the antiplasmodial properties of *Phyllanthus niruri* resident in its chloroform fraction. Asian Pac J Trop Med.

[6] Feng Z, Zhang JT (2004). Melatonin reduces amyloid beta-induced apoptosis in pheochromocytoma (PC12) cells. J Pineal Res.

[7] Thorn DC, Meehan S, Sunde M (2005). Amyloid fibril formation by bovine milk kappa-casein and its inhibition by the molecular chaperones alphaS-and beta-casein. Biochemistry.

[8] Onoue S, Ohshima K, Endo K, Yajima T, Kashimoto K (2002). PACAP protects neuronal PC12 cells from the cytotoxicity of human prion protein fragment 106–126. FEBS Lett.

[9] Xing QL, Chen JW, Ma RQ (2008). Protective effect of panaxatriol saponins on cerebral ischemia. J Sun Yat-sen Univ (Medical Sciences).

[10] Yang HJ, Wei CY (1999). The clinical approach of ginseng chemical composition. Renshen Yanjiu.

[11] Tan RJ (2000). Tan RJ: The research progress on pharmacological effects of *Polygonum multiflorum*. Anthology of Medicine.

[12] Li GS, Tian JW, Feng FH, Yang JQ (2006). Protective effect of ginsenoside Rb3 from rat cortex mitochondrial injuries due to cerebral ischemia. Chin J New Drug.

[13] Dai CF, Yang XY (1999). The study on recent development of *Fructus Ligustri Lucidi*. Shanxi J Tradit Chin Med.

[14] Cheng SY, Chen YB, Wang Q (2010). The experimental research on protective effect of Bu-Shen-Yi-Zhi compound in Aβ25–35 injured PC12 cells. J N Chin Med.

[15] Fang CW (2000). The study on Harvesting and production processing method of *Cortex Moutan*. J Chin Med Mat.

[16] Qiao ZL, Guo L, Li F (2009). The effects of Bu-Shen-Yi-Zhi compound on central neurotransmitter in Alzheimer’s disease model rats. Chin Arch Tradit Chin Med.

[17] Li FJ (1999). The medical food aplication of barbary wolfberry fruit. Lishizhen Med Materia Medica Res.

[18] Zhong ZG, Liu MC, Lai SL, Gao J, Cheng SY (2005). The effects of Bu-Shen-Yi-Zhi compound on central neurotransmitter in Alzheimer’s disease model rats. Chin J Clin Rehabil.

[19] Cheng SY, Zhong ZG, Liu MC, Lai SL (2003). The experimental research on protective effect of Bu-Shen-Yi-Zhi compound in AD cell model. Chin Med Res.

[20] Zhang KH, Lai SL, Wang Q, Cheng SY, Chen YB (2002). The effects of Bu-Shen-Yi-Zhi compound on Space exploration learning and memory function in AD animal model. Chinese Traditional and Herbal Drugs.

